# Experiences of adolescents seropositive for HIV/AIDS: a qualitative study

**DOI:** 10.1016/j.rppede.2015.08.019

**Published:** 2016

**Authors:** Eliana Galano, Egberto Ribeiro Turato, Philippe Delmas, José Côté, Aida de Fátima Thomé Barbosa Gouvea, Regina Célia de Menezes Succi, Daisy Maria Machado

**Affiliations:** a Escola Paulista de Medicina, Universidade Federal de São Paulo (EPM-Unifesp), São Paulo, SP, Brazil; b Faculdade de Ciências Médicas, Universidade Estadual de Campinas (Unicamp), Campinas, SP, Brazil; c Institut et Haute Ecole de la Santé La Source (HES-SO), Lausanne, Switzerland; d Université du Québec à Montréal (UQAM), Montreal, Canada

**Keywords:** HIV infection/psychology, Life-changing events, Qualitative research, Adolescent

## Abstract

**Objective:**

Explore the meanings attributed by young individuals about "living as an adolescent with HIV" in a group of patients that acquired the infection at birth and the elements involved with the adherence to antiretroviral treatment.

**Methods:**

Qualitative study, involving 20 subjects (aged 13-20 years), followed at services specialized in the treatment of pediatric AIDS in São Paulo, Brazil. Semi-structured interviews were carried out of which script consisted of questions about their personal histories, experiences and difficulties they must face while living with HIV/AIDS.

**Results:**

Being "normal" and "different" were central issues voiced by the participants. However, a normal life situation is guaranteed by being responsible with one's health, the condition that the diagnosis be kept secret and concerns about HIV transmission and dissemination to a sexual partner. The answers about treatment show that adherence is a dynamic process and involves moments of greater or lesser interest in relation to care for one's health. The adolescents have plans and projects and although HIV is considered a stressor, positive perspectives for the future prevailed.

**Conclusions:**

To live as an adolescent with HIV involves subtle dimensions that need to be recognized and legitimized by professionals who follow the trajectory of these young individuals. It is necessary to allow a space in which the adolescents can reflect and find support regarding issues related to the construction of their sexuality and care of one's own body.

## Resumo

**Objetivo:**

Explorar os significados atribuídos pelos jovens a "viver a adolescência com o HIV" em um grupo de pacientes que adquiriu a infecção ao nascimento e os elementos implicados na adesão ao tratamento antirretroviral.

**Métodos:**

Pesquisa de natureza qualitativa, com 20 sujeitos (13 a 20 anos), acompanhados em serviços especializados no tratamento da Aids pediátrica em São Paulo, Brasil. Foram feitas entrevistas semidirigidas cujo roteiro foi composto por questões sobre suas histórias pessoais, dificuldades e experiências que enfrentam diante da infecção pelo HIV/Aids.

**Resultados:**

Ser "normal" e "diferente" foram questões centrais no discurso dos participantes. Entretanto, a condição de uma vida normal é garantida mediante a responsabilidade com a saúde, a ressalva de que seja mantido o segredo do diagnóstico e as preocupações com a transmissão do vírus e divulgação do HIV ao parceiro sexual. As respostas sobre o tratamento apontam que a adesão é um processo dinâmico e envolve momentos de maior ou menor interesse em relação aos cuidados com a saúde. Os adolescentes têm planos e projetos e, apesar de o HIV ser considerado um agente estressor, prevaleceram perspectivas positivas diante do futuro.

**Conclusões:**

Viver a adolescência com o HIV envolve dimensões delicadas, que necessitam ser reconhecidas e legitimadas pelos profissionais que acompanham a trajetória desses jovens. Trata-se de possibilitar um espaço no qual o adolescente possa refletir e encontrar apoio para as questões relacionadas à construção de sua sexualidade e cuidados com seu próprio corpo.

## Introduction

In the third decade of the human immunodeficiency virus (HIV) epidemic, health professionals, researchers and caregivers are faced with the first generation of young individuals who became infected through vertical transmission. Over the years, one sought to ensure access to treatment that would allow increased life expectancy of these children and contemplates their psychosocial needs. The appalling demands of a newly described disease did not allow the professionals who treated these children to suspect that they could reach adolescence, with all the challenges that are associated with it. This population group has distinct characteristics from adult or young individuals that contracted the disease during adolescence.^1^ Many of them have lost their parents as a result of the human immunodeficiency syndrome (AIDS - Acquired Immune Deficiency Syndrome), which results in early bereavement, disruption of affective bonds and family rearrangements.^2^

In addition to adolescence, a stage of life permeated by changes, discoveries, search for identity and autonomy, they have a disease full of stigmatizing attributes and the complex legacy of secrets involving families affected by HIV. Qualitative studies have identified that the main difficulties reported by HIV-positive young individuals included diagnostic disclosure to third parties, interpersonal relationships, adherence to treatment and the psychological burden of living with a chronic illness associated with death, prejudice and social exclusion.^3^^,^ 4 Research suggests that the challenges related to HIV-infected adolescents are constant and therefore need to be identified and further investigated.^5^^,^ 6

For professionals seeking to care for their patients in all their dimensions, it is critical to identify the particularities, desires and difficulties, from the perspective of the adolescents themselves. The objective of this study was to explore, through the qualitative methodology, the meanings attributed by young individuals to the phenomenon of "living the adolescence with HIV" in a group of patients who acquired the infection through vertical transmission and the elements involved in the adherence to the antiretroviral treatment.

## Method

Qualitative research, carried out through interviews with open questions, comprising situations found in the experiences of young individuals in the presence of HIV/AIDS: (1) Life history; (2) Adolescence and seropositivity and (3) Health care and treatment.

The population of this study comprised 20 adolescents, selected from a group of 268 participants in the longitudinal study Adoliance, an international cooperation project for the study of psychosocial factors related to the life experiences of HIV-positive young individuals, which started in April 2009. In this phase of the work, carried out between November and December 2011, we included subjects aged 13-20 years, enrolled in four services that provide care in pediatric AIDS, located in São Paulo, Brazil. The choice of candidates was intentional and we sought to diversify the group by including boys and girls, at least one from each of the participating centers, at different ages and a history of compliance or non-compliance with the antiretroviral treatment. To attain subject enrollment, professionals from different departments identified the adolescents that did not have difficulties to verbalize their needs and experiences of being an HIV-positive adolescent.

The meetings with the young individuals were previously scheduled and carried out in a private place. The discontinuation of new subject enrollment occurred by theoretical saturation, that is, when we observed a redundancy in the speech in the last interviews, so that the adding of new information would not alter the understanding of the obtained material.^7^^,^ 8

The interviews were recorded with the approval/consent of the participants and tutors and verbatim transcribed. As an option, in order to facilitate data organization, the Nvivo 9 software (Qualitative Solutions and Research Pty Ltd, Australia, 1994) was used.

The researchers used a theoretical framework based on the basic concepts of health psychology, comprising its contextual determinants and adaptive responses to adverse situations in the participants' life experiences. Thus, during several exploratory data analyses, categories for discussion emerged from the meaning core of the performed interviews. To ensure the accuracy of the analysis process, the material content was independently coded by three researchers and subsequently validated by an experienced professional in qualitative interview encoding.

The study was approved by the regulating agencies of each of the collaborating centers and the National Research Ethics Committee (Conep), N. 15693. All participants who were 18 years and the tutors of those younger than 18 years old signed the informed consent form after being informed about the benefits and possible risks of the study. In the case of those younger than 18 years, in addition to the legal tutors, the respondents also read and signed the informed consent form.

## Results

The sample consisted of 11 girls and 9 boys with a mean age of 17 years. Table 1 describes the sociodemographic characteristics of adolescents who participated in this phase of the study.

**Table 1:** Distribution of sociodemographic characteristics of adolescents.

Characteristics	Population (*n*=20)
*Gender*	
Female	11
Male	09
	
*Age range (years)*	
13-15	08
16-18	08
19-20	04
	
*Educational level (years of schooling)*	
5-8	07
9 or more	13
	
*Ethnicity/skin color (self-reported)*	
White	08
Black	01
Mixed-race	11
	
*Religion*	
Yes	18
No	02
	
*Lives with*	
Father and/or mother	12
Other relatives	06
Non-family members	01
Institution	01
Father	04
Mother	08
	
*Orphaned*	
Father and mother	03

The analysis of the material from the interviews allowed the development of two major categories with six sub-categories, as shown in Table 2. The category "Adolescence and seropositivity"' is related to the main experiences of being a seropositive adolescent for HIV/AIDS, originating the subcategories that involved "being normal" and "being different", to "living with the silence of the diagnosis" and the disease implications on "affective and sexual relationships". The second category refers to health care and treatment and includes subcategories associated with the main obstacles, motivations and desired interventions.

**Table 2:** Categories and subcategories created based on the analysis of the interviews.

Categories	Subcategories
Adolescence and seropositivity	To be normal X to be different
	Living with the silence of the diagnosis: from the recipient to the secret bearer
	Affective and sexual relationships

Health care and treatment	Obstacles to treatment: the objective and subjective dimension
	Motivation and treatment management
	Desired interventions

### Adolescence and seropositivity

"Being normal" and "being different" were central issues that permeated the speech of the study participants. The reference to normality was demonstrated by narratives that compare their daily lives to other adolescents who do not live with chronic diseases: they work, study, go out and interact with family and friends.

*Normal, it does not change anything! It's the same thing. I play ball, I walk, I do everything! It's too good... (Male, 14)*

However, the condition of "having a normal life" is guaranteed by the responsibility of health care and the condition that the diagnosis must be kept in secret.

*They do not know about my problem, never! Everyone thinks I'm a perfect person... When you take treatment seriously, you can lead a normal life. (Male, 18 years)*

Sometimes mentioning the difference is clearly accepted by respondents and is based, particularly, on the restrictions imposed by living with a chronic disease, such as the use of medications and frequent medical consultations.

*You are different from the others, you will take medicine, you will undergo a treatment and go to the doctor for the rest of your life... you feel different because you have a secret! (Female, 14)*

For most participants in this study, "coexistence and maintaining the secret" regarding HIV is something that was unquestioningly incorporated into their lives. It concerns the private life domain and talking about the virus can bring discomfort, embarrassment and risk of rejection due to the stigma associated with HIV. In this sense, several phrases by the respondents reproduce these messages that were learned throughout life.

*I never had the courage to tell, there is no need, I learned that! (Female, 18 years)*

The narratives of respondents show that the knowledge about HIV remains reserved for the nuclear family or trusted relatives and very close friends. Still, issues related to the disease are not shared even among those who know about the infection.

[Who knows about HIV?] *My mom and dad, no one else! For them and for me it is like if I did not have the disease! (Male, 18 years)*

In the case of "romantic relationships", young individuals describe strong concerns about the transmission of infection to the sexual partner, even among those who have not yet had romantic or sexual experiences. However, there was a consensus that having sex must be done responsibly and with extreme care, considerations that are not shared with the people close to them.

*When you think you are going to have sex, you are afraid of transmitting the HIV*. [Do you think too much about it?] *Everyone who has HIV thinks about it! (Female, 15 years)*

From the perspective of the participants, the confidentiality surrounding the disease is no longer compatible in the context of loving relationships and, regardless of age or gender, they consider that the disclosure of their seropositivity must be made sometime during the relationship. However, to know the best time to do it and decide who they should trust to tell are questions accompanied by anxieties and worries. The main reported difficulties are related to the fear of abandonment due to prejudice and the belief that the secret, when entrusted to another person, might not be kept by this individual over time.

*To tell your boyfriend, You have to be with him for some time, make sure he loves you... if people knew, they would probably not stay with me. (Female, 20)*

### Health care and treatment

The biggest discomfort ("obstacle") to the treatment is related to the inflexibility regarding the medication schedule, which imposes restrictions to activities such as trips or parties with friends. Frequently, the sleep cycle of the adolescents is interrupted, as they need to get up early or sleep late to take the medication.

*On vacations or when I don't have classes, I have to wake up at 3:30 to take the medication and then I can't go back to sleep ... (Male, 17 years)*

Undesirable effects such as nausea, stomach pain, malaise and bad taste and amount of ingested tablets/pills, were reported as barriers to good treatment adherence.

Depressive moods, irritability, anxiety, stress, family conflicts and feelings of anger caused by not accepting the disease were also associated with missed doses or drug treatment discontinuation.

*I feel angry, very angry with my life and with everyone. You know, if it is for me to die, I will die soon... My life is like two separate things...* [Separate how?] *By being aware of what is like having HIV. (Female, 17)*

Table 3 shows the summary of the main obstacles to treatment adherence. Fear of becoming ill, which is connected to the desire of having a healthy lifestyle in order to work, study and make plans, was the main "motivation" mentioned by the participants to adhere to treatment.

**Table 3:** Health care and treatment - obstacles.

Obstacles	Obstacles
Objective dimension	Subjective dimension
Interference with daily activities: sleep disruption	Depressive moods, irritability, nervousness and stress
Undesirable effects: nausea, stomach pain and malaise	Feelings of rebellion and non-acceptance of the disease
Medication characteristics: palatability and number of ingested tablets	Family conflicts
Belief that antiretroviral drugs cause health hazards	Taking the medication is a reminder of the HIV-status, a condition they wish to forget

In some cases, the need to obtain approval by doctors, relatives or boyfriends/girlfriends are other factors that contributed to a good treatment adherence. Similarly, the recognition of its importance to reduce the risk of transmission, especially to sexual partners, showed strong influence on continuity of care.

*It is good when you have a doctor treating you since you were small, who you trust. This motivates me to never think about stopping... (Female, 15 years)*

Finally, among the "desired interventions", the adolescents qualify the guidance, the explanations and information that are routinely provided by the service professionals as positive.

The close contact and the trust established with the team that accompanies them from early childhood were mentioned as key aspects to a good response to the adherence. The importance of group activities or spaces to meet other people living with HIV were also emphasized by respondents, as it encourages discussion and coping with experiences involving the HIV-positive adolescent.

*I think it's cool to have lectures, groups. I wanted to know a lot of people who have HIV! (Female, 16 years)*

Moreover, they want to have updated knowledge on the scientific advances and suggest that issues be addressed that cover future concerns, such as marriage and ways to prevent HIV transmission to people who live with them.

*Prevention, medication, family... They are the most important issues. To talk, learn more about the scientists who are seeking the cure... (Male, 17 years)*

While for some young people, motivate, insist and remember the medications are expressions of concern and affection, for others, the insistence of professionals for perfect adherence causes discomfort and feelings of being disrespected regarding their rights of transitory interruption or not of the treatment. There are times when they would rather lie to avoid the reactions of irritation or disappointment by doctors and relatives (Table 4).

**Table 4:** Health care and treatment - motivations and interventions.

Motivations	Suggested interventions
Fear of becoming ill	Updated information
To have accompanied former negative experiences	Group activities and individual psychological support
Recognize their role in reducing transmission risks	Consultations to strengthen and encourage treatment without an overbearing attitude
Need to obtain approval

*Give us a break, because it is a lot of pressure... The doctor, the psychologist pushing us, saying that you have to take it, or you'll die. Also try very hard to understand... Why, why? Because I do not want to get in touch with reality, because I'm afraid! (Male, 17 years)*

## Discussion

This research helped us to understand the psychosocial experiences of the first generation of young individuals who acquired HIV through vertical transmission, through reports of their life stories and obstacles faced in the context of living with a chronic disease, in order to prepare health professionals to better care for their patients.

In the several dimensions of daily life, such as participation in social activities, school and work, the adolescents' speech make references to a normal life, that is, common or equal to other young individuals who are not infected. The effort of HIV-positive adolescents in searching for normality has been described by different researchers who studied the needs of young individuals living with HIV.^9^^,^ 10

As can be observed, the perception of normality and the feeling of equality are justified by the absence of symptoms or physical characteristics of a diseased body, factors that, in turn, can negatively influence treatment adherence.^10^ However, feelings of ambivalence are observed when these young individuals are faced with the need to undergo care that involves an endless treatment and routine medical consultations. Another exception regarding normality is related to living with the secret of the diagnosis and the possibility of transmitting the virus, findings that are also consistent with other studies.^5^^,^ 9

An important issue that deserves special attention concerns the dynamics of the secrecy surrounding patients infected by HIV/AIDS. The disclosure of the diagnosis to others is not a simple process, because revealing one's HIV status can leave the individual vulnerable to social stigma, prejudice and discrimination, aspects that are widely identified in the adult population living with HIV/AIDS.^4^ The results of this study suggest that the silence about the HIV-positive status remains restricted to families who live with the infection and, from the point of view of respondents, the reasons that justify this behavior were associated with fear of prejudice, rejection and social isolation.^11^^-^ 14 Nevertheless, the adolescents' speech shows that the disclosure of HIV diagnosis should be shared at some point, with current or future sexual partners, a decision-making that is surrounded by fears and ambivalence. Even in the absence of clear and defined parameters for the disclosure of the infection, it is necessary to trust someone to talk about it, to be assured that the other will keep the secret and, in some situations, use some type of survey to identify the concepts their peers have on the disease.^15^

When it comes to matters of sexuality and romantic relationships, in addition to the challenge of disclosing one's HIV status to a sexual partner, there are legitimate strong concerns about transmission of the virus to the partner, also mentioned by other authors.^4^^,^ 16 ^,^ 17 Still in the context of romantic relationships, desires and anxieties caused by the HIV status are mixed up and, sometimes, avoidance behaviors prevail in the presence of affective approaches, as observed in several publications.^4^^,^ 18

Regarding health care and treatment, the results of this study suggest that adherence to the medication regimen is a dynamic process, and involves subjective and objective dimensions and moments of greater or lesser interest in relation to clinical monitoring. Despite the weariness regarding the use of medications and sometimes the desire to discontinue the treatment, most of the young individuals in this study showed to be aware that following the prescribed recommendations is a necessary condition to maintain good quality of life. The findings of this study were consistent with other studies, which described that adherence is favored by the belief in medication efficacy^19^ and when health care becomes a priority in their lives. The acceptance of the HIV status and acknowledgment of death, which come with the maturity, were also decisive factors for maintaining adequate treatment. The connection between patient and health care professional and family support promote the motivation to face the health and disease processes.^20^ On the other hand, some adolescents do not like to feel pressured and consider that overbearing attitudes by caregivers violate their rights and autonomy to make decisions on the treatment. As for the barriers and obstacles to full adherence, the participants mentioned the adverse effects of the drugs and their interference in daily activities. In the subjective dimension, moments of sadness, stress or even the simple desire to living life without reminding oneself of the virus justify interruptions, even if momentary, of the treatment.

Regarding the desired interventions, young individuals stated that they are satisfied with the care received in the pediatric setting, surrounded by attention and demonstrations of affection from professionals. Nevertheless, they want more detailed information on ways to prevent transmission of the virus, with discussion of topics related to technological advances, as well as the dilemmas experienced by young individuals in the context of seropositivity. In some cases, the group activities are recommended, because they favor the identification with peers and thus, the maintenance of the feeling of normality.

This study has limitations because its results are not generalizable and partially show the complexity of the process of living adolescent experiences in the context of HIV-positive status. Longitudinal studies are recommended to explore the social conditions and emotional risks involved in this trajectory. In addition, the qualitative methodology is still little familiar to the scientific community and pediatricians involved with the care of these children and adolescents.

It can be concluded from the reports that "living as an adolescent with HIV" involves delicate dimensions, such as silence and secrets, search for normality, finding the differences, virus transmission dilemmas and also the drug treatment management. Recognizing these aspects can be used to guide the work of the multidisciplinary team, especially pediatricians, whose care transcends the physical control of the disease and viral replication.
